# Identification of Leukocyte telomere length-related genetic variants contributing to predisposition of Esophageal Squamous Cell Carcinoma

**DOI:** 10.7150/jca.45165

**Published:** 2020-06-23

**Authors:** Ziqiang Li, Yemei Song, Yeyang Xu, Yue Shen, Nasha Zhang, Ming Yang, Dianke Yu

**Affiliations:** 1 Department of General Surgery, The First Affiliated Hospital of Shandong First Medical University, Jinan, Shandong Province, China.; 2 Shandong Provincial Key Laboratory of Radiation Oncology, Cancer Research Center, Shandong Cancer Hospital and Institute, Shandong First Medical University and Shandong Academy of Medical Sciences, Jinan, Shandong Province, China.; 3 Department of Radiation Oncology, Shandong Cancer Hospital and Institute, Shandong First Medical University and Shandong Academy of Medical Sciences, Jinan, Shandong Province, China.; 4 School of Public Health, Qingdao University, Qingdao, Shandong Province, China.

**Keywords:** telomere, ESCC, CXCR4, TERT, OBFC1

## Abstract

**Background:** Cancers may arise from cells with dysregulated telomeric functions due to shorten telomere length. We and others previously found that short leukocyte telomere length was associated with markedly evaluated risk of esophageal squamous cell carcinoma (ESCC). Hence, we hypothesized that single nucleotide polymorphisms (SNPs) associated with shorter telomere length may contribute to ESCC predisposition.

**Methods:** We systematically evaluated association between seven candidate seven SNPs (*CXCR4* rs6430612, *TERT* rs13172201, *TERT* rs10069690, *TERT* rs2853676, *TERT* rs451360, *OBFC1* rs4387287, and *VPS34* rs2162440) and ESCC risk in two case-control sets consisting of 1588 ESCC cases and 1600 controls. Logistic regression models were utilized to estimate associations between SNPs and ESCC susceptibility and odds ratios (ORs) and their 95% confidence intervals (95% CIs) were computed.

**Results:** We firstly identified three SNPs (rs6430612, rs13172201 and rs4387287) which are significantly associated with telomere length in Chinese populations (all *P*<0.05). Importantly, *CXCR4* rs6430612 and *OBFC1* rs4387287 polymorphisms significantly confer reduced risk of ESCC (*P*=1.7×10^-7^ and *P*=3.9×10^-5^). On the contrary, we observed an evidently increased risk for ESCC development associated with *TERT* rs13172201 genetic variant (*P*=2.2×10^-4^).

**Conclusions:** In summary, rs6430612, rs13172201 and rs4387287 might be key genetic components in complicated regulation of telomere length and contributing to ESCC predisposition. Our results elucidate the prevalent involvement of genetic variants in telomere biology and further provide pathogenic insights into the role of telomeres in cancer development.

## Introduction

In 2018, GLOBOCAN estimates that there were 572,034 new esophageal cancer cases and 508,585 deaths in the world 1. Eastern Asia showed the highest region-specific incidence age-standardized rates in both males and females for esophageal cancer (17.9 and 6.8), which are much higher than other regions such as Western Europe (6.8 and 1.7) and North America (5.5 and 1.5) 1. In Eastern Asia, the majority of esophageal cancer cases were diagnosed as esophageal squamous cell carcinomas (ESCC) but not esophageal adenocarcinoma 1-3. There are major risk factors for ESCC, i.e. heavy drinking and smoking as well as dietary components including nutritional deficiencies and nitrosamines 2-4. However, the full etiology of ESCC has yet to be elucidated. In recent years, genome-wide association studies (GWAS) identified a few susceptibility gene loci for ESCC, elucidating that genetic components also contribute to ESCC development 5-14.

Telomeres locating at the ends of linear chromosomes maintain integrity of human genome in cells 15. Cell divisions lead to inevitable erosion of linear chromosomes. However, telomerase could promote telomere lengthening and telomere length homeostasis in stem cells and malignant cells 15-17. Multiple studies indicate that telomere length in peripheral blood lymphocytes was significant associated with cancer susceptibility 18-23. Either extreme short leukocyte telomere length or extreme long leukocyte telomere length may contribute to cancer development. Genomic instability, a hallmark of cancer, occurs in cells with shorten telomeres and, thus, individuals with extreme short leukocyte telomeres may confer to elevated risk of several cancers such as ESCC 17,24,25.

The heritability estimates of human telomere length are 44%~80%, suggesting the key role of genetic factors in controlling telomere length 26,27. Several quantitative trait linkage (QTL) GWAS and candidate gene QTL studies have mapped various SNPs correlated to leucocyte telomere length 28-37. Several telomere length-related SNPs have been previously found to confer susceptibility of cancers including ESCC. For instance, we previously identified two SNPs (rs621559 and rs398652) which are significantly associated with telomere length in Chinese as well as ESCC predisposition 24. However, it is still largely unclear how recent GWAS identified telomere length-related SNPs impact ESCC development. Following these leads we performed an in-depth analysis of the genetic variability of these telomere length-related SNPs in ESCC.

## Material and Methods

### Study case-control sets

In the current study, there are two case-control sets, Jiangsu discovery set and Shandong validation set (Supplementary Table 1). The detailed information on subject recruitments has been reported in details previously 38. All subjects enrolled in the study were ethnic Han Chinese. This study was approved by the Institutional Review Boards. At recruitment, the written informed consent was obtained from each subject. The methods were carried out in accordance with the approved guidelines.

### Measurement of leukocyte telomere length

As reported previously, telomere length of leukocytes was detected using quantitative real-time PCR 39. All quantitative real-time PCR reactions were performed on ABI QuantStudio 6 Flex Real-Time PCR System (Foster City, CA, US). The relative leukocyte telomere lengths were calculated as the ratio of the telomere repeat copy number and the *β-globin* gene copy number (the T/S ratio).

### SNPs selection and genotyping

In a previous study, we identified seven SNPs (*CXCR4* rs6430612, *TERT* rs13172201, *TERT* rs10069690, *TERT* rs2853676, *TERT* rs451360, *OBFC1* rs4387287, and *VPS34* rs2162440) are significantly or marginally associated with telomere length of leucocytes in Shandong heathy subjects (all *P* < 0.10). 40.In this study, these SNPs were firstly genotyped in the controls of Jiangsu discovery set using the iPLEX Sequenom MassARRAY platform (Sequenom Inc., San Diego, CA, USA) as reported previously 40. Telomere length-related* CXCR4* rs6430612, *TERT* rs13172201, and *OBFC1* rs4387287 SNPs were then analyzed in both discovery and validation case-control sets. A 15% random sample was reciprocally tested and the reproducibility was 100%.

### Statistics

The differences in demographic variables, smoking status, drinking status between patients and controls were examined using Pearson's χ^2^ test. The association between relative telomere lengths of leukocytes and the SNPs were calculated using multivariable linear regression adjusted for age and sex. The associations of *CXCR4* rs6430612, *TERT* rs13172201, and *OBFC1* rs4387287 with ESCC risk were estimated by odds ratios (ORs) and their 95% confidence intervals (95% CIs) computed by logistic regression models. All ORs were adjusted for age, sex, smoking or drinking status, where it was appropriate. A *P* value of less than 0.05 was used as the criterion of statistical significance. Bonferroni correction was used for multiple comparisons. All statistical tests were two-sided. All analyses were performed using SPSS 16.0 (SPSS Inc.).

## Results

### Identification of SNPs significantly associated with telomere length

Our previous study indicated that *CXCR4* rs6430612, *TERT* rs13172201, *TERT* rs10069690, *TERT* rs2853676, *TERT* rs451360, *OBFC1* rs4387287, and *VPS34* rs2162440 are significantly or marginally associated with telomere length of leucocytes. As a result, we firstly validated the association of these genetic polymorphisms with telomere length in heathy subjects of Jiangsu set. As shown in Table 1, we observed significant correlations between *CXCR4* rs6430612, *TERT* rs13172201, and *OBFC1* rs4387287 SNPs and telomere length (all *P* < 0.05). In details, the minor alleles of *CXCR4* rs6430612 and *OBFC1* rs4387287 genetic variations are remarkably associated with long telomere length. On the contrary, the *TERT* rs13172201 T allele is associated with markedly short leukocyte telomere and showed to be risk allele. However, we did not find that *TERT* rs10069690, *TERT* rs2853676, *TERT* rs451360 and *OBFC1* rs4387287 polymorphisms are significantly associated with telomere length of leukocytes in Jiangsu set (all *P* > 0.05) (Table 1).

### Association between rs6430612, rs13172201 and rs4387287 SNPs and ESCC risk

To explore the impact of telomere length-related *CXCR4* rs6430612, *TERT* rs13172201 and *OBFC1* rs4387287 polymorphisms on ESCC susceptibility, we next genotyped the SNPs in both Jiangsu and Shandong sets. Unconditional logistic regression analyses showed that the odds of having the *CXCR4* rs6430612 CT genotype in ESCC patients was 0.40 (95% CI = 0.25-0.62, *P* = 4.7×10^-5^) compared with carriers of the CC genotype in the Jiangsu set (Table 2). Importantly, individuals with the *CXCR4* rs6430612 CT genotype showed 43% decreased risk to develop ESCC compared with those with the *CXCR4* CC genotype in Shandong set (95% CI = 0.42-0.78, *P* = 4.0×10^-4^) (Table 2). Pooled analyses indicated that the odds of having the *CXCR4* rs6430612 CT genotype in ESCC patients was 0.51 (95% CI = 0.40-0.66, *P* = 1.7×10^-7^, still statistically significant after Bonferroni corrections) compared to the CC genotype (Table 2).

Logistic regression analyses elucidated that the *TERT* rs13172201 T allele was ESCC risk allele. Individuals carrying the CT or TT genotype had an OR of 1.42 (95% CI = 1.08-1.88, *P* = 0.012) or 1.44 (95% CI = 0.93-2.21, *P* = 0.102) for developing ESCC, respectively, compared with individuals having the CC genotype in the discovery set (Table 3). In line with this, carriers of *TERT* rs13172201 CT or TT genotype showed 1.33-fold elevated risk to develop ESCC compared with carriers of the CC genotype (95% CI = 1.08-1.64, *P* = 0.007; OR = 1.52, 95% CI = 1.14-2.02, *P* = 0.004) in Shandong set (Table 3). Pooled analyses all support the hazardous role of the *TERT* rs13172201 T allele in ESCC (CT genotype: OR = 1.37, 95% CI = 1.16-1.61, *P* = 2.2×10^-4^; TT genotype: OR = 1.50, 95% CI = 1.19-1.91, *P* = 0.001, still statistically significant after Bonferroni corrections) (Table 3).

In Jiangsu set, a 0.74-fold reduced risk of developing ESCC was associated with the *OBFC1* rs4387287 CA genotype (95% CI = 0.55-0.99; *P* = 0.049) compared with the CC genotype. Consistently, there was a 0.67-fold (95% CI = 0.54-0.84, *P* = 3.9×10^-4^) or 0.64-fold (95% CI = 0.44-0.95, *P* = 0.025) decreased ESCC risk was observed among carriers of the *OBFC1* rs4387287 CA or AA genotype compared to individuals with the CC genotype in Shandong set (Table 4). In the pooled analyses, similar results were observed (the CA genotype: OR = 0.67, 95% CI = 0.58-0.82, *P* = 3.9×10^-5^; the AA genotype: OR = 0.59, 95% CI = 0.43-0.82, *P* = 0.002, still statistically significant after Bonferroni corrections) (Table 4).

### Stratified analyses of association between rs6430612, rs13172201 and rs4387287 SNPs and ESCC risk

ESCC genetic susceptibility associated with telomere length-related *CXCR4* rs6430612, *TERT* rs13172201 and *OBFC1* rs4387287 polymorphisms was further investigated by stratifying for age, sex, smoking and alcohol drinking status using pooled data of two case-control sets (Table 5). For the *CXCR4* rs6430612 polymorphism, a decreased risk of ESCC was associated with the CT or TT genotype in both individuals aged older than 66 years and ones aged 66 years or younger (both *P* < 0.05). Similarly, in stratified analyses with sex, smoking or alcohol drinking status, the *CXCR4* rs6430612 genetic variant was significantly associated with reduced risk in males, females, smokers, nonsmokers, drinkers or nondrinkers (all *P* < 0.05) (Table 5).

For the *TERT* rs13172201 SNP, a significantly elevated risk of ESCC associated with the CT or TT genotype was observed among nonsmokers (OR = 1.82, 95% CI = 1.41-2.33, *P* = 2.8×10^-6^), but not among smokers (OR = 1.20, 95% CI = 0.97-1.49, *P* = 0.090). There was statistically significant gene-smoking interaction for the *TERT* rs13172201 SNP (*P*_interaction_ = 0.024). In stratified analyses with age, sex or alcohol drinking status, the *TERT* rs13172201 polymorphism was markedly associated with increased risk in all sub-groups (all *P* < 0.05). No significant gene-environment interactions were observed (Table 5, Table 6).

For the *OBFC1* rs4387287 polymorphism, stratified analyses showed that significantly decreased ORs for ESCC development were only observed in males (OR = 0.57, 95% CI = 0.47-0.70, *P* = 2.3×10^-7^), smokers (OR = 0.44, 95% CI = 0.35-0.55, *P* = 1.5×10^-7^), and alcohol drinkers (OR = 0.40, 95% CI = 0.32-0.52, *P* = 7.6×10^-8^). A statistically significant gene-sex, gene-smoking or gene-drinking interaction was observed (*P*_interaction_ = 0.001, 0.002 or 2.2×10^-4^). However, there was no significantly decreased risk for females, nonsmokers or nondrinkers with the *OBFC1* rs4387287 CA or AA genotype compared with ones with the CC genotype. Evident association between the rs4387287 CA or AA genotype and ESCC susceptibility was found both individuals aged older than 66 years and ones aged 66 years or younger (both *P* < 0.05) (Table 5, Table 6).

## Discussion

Due to incomplete replication of the 3' end of each DNA strand, gradual shrink of telomeres after every mitotic division would lead to chromosomal instability of somatic cells. Genome instability caused by shorten telomere length will finally promote oncogenic phenotypes and malignant transformation of normal cells. Considering short telomere length of leukocytes has been associated with increased ESCC susceptibilities 24,25, we hypothesized that germline genetic variants associated with shortened telomeres may contribute to increased ESCC risk. To test this notion, in the present study, we systematically evaluate thirty candidate genetic variants reported previously and firstly identified three SNPs associated with telomere length in Chinese. Based on analyzing 1,588 ESCC cases and 1,600 controls, we found that *CXCR4* rs6430612 and *OBFC1* rs4387287 polymorphisms significantly confer reduced risk of ESCC. On the contrary, an evidently increased OR for ESCC development was associated with *TERT* rs13172201 genetic variant.

We previously found that *CXCR4* rs6430612 polymorphism is a telomere length-related SNP contributing to GCA risk, with the T allele as a protective allele 40.CXCR4 is a chemokine receptor whose expression was significantly correlated with invasion, angiogenesis, metastasis, and prognosis of ESCC 41-43. For instance, expression of CXCR4 in ESCC is of major relevance in a German population 41. High expression levels of CXCR4 in cytoplasm and nuclei were associated with poor cause-specific survival in Japanese 43. In a Chinese population, the expression of CXCR4 in tumor cells was positively associated with tumor status and clinical stage 42. In addition, ESCC cells coexpressing CD133 and CXCR4 possess the characteristics of cancer stem cells and contribute to poor prognosis of patients 44. The crucial involvement of CXCR4 in ESCC development may illuminates the significant association between the rs6430612 polymorphism and ESCC risk.

The *TERT* gene locating at chromosome 5p15.33 encodes the catalytic subunit of telomerase reverse transcriptase. TERT is a key component of the RNA-protein complex which functions in maintaining telomere ends. Accumulated evidences indicate that *TERT* SNPs contribute to ESCC risk 45-47. For the rs13172201 polymorphism, Wang et al identified it as one of five *TERT* independent risk loci across different cancer types (*P* = 0.041 and *P*_Conditional_ = 2.04 × 10^-6^) based on sequential conditional analyses 48. Consistent with this, we demonstrated that a significantly elevated ESCC risk was associated with minor alleles of the rs13172201 variant.

The *OBFC1* gene, also known as *STN1*, codes a protein which is a subunit of a telomere-associated complex including C17ORF68 and TEN1 49. OBFC1 is also one of the components of an alpha accessory factor that promotes the activity of DNA polymerase-alpha-primase, the enzyme that initiates DNA replication. Importantly, human* OBFC1* locus genetic variants are involved in telomere biology 50 and confer risk of melanoma, epithelial ovarian cancer, thyroid cancer, uterine leiomyoma and pancreatic cancer 51-56. However, several cancer susceptibility *OBFC1* polymorphisms in Caucasian populations, such as rs7902587, rs2487999 and rs9420907, do not exist in Han Chinese populations except rs4387287. Strikingly, we found that the *OBFC1* rs4387287 variant significantly contributes to ESCC risk. In the stratified analyses, we observed a significant gene-smoking or gene-drinking interaction for rs4387287. The exact mechanisms for these gene-environment interactions are currently unknown. However, a systematic review of 84 studies and meta-analysis indicated that shorter telomeres were found among ever smokers compared to those who never smoked 57, which may contribute at least in part to our stratified results. Together, these results add new lines of evidences highlighting the role of *OBFC1* in telomere-related malignant transformation.

There are several limitations in the current study. First of all, inherent selection bias may exist since this hospital-based study enrolled ESCC cases and healthy controls from hospitals. Therefore, it is important to validate these findings in a population-based prospective study. Second, the sample size of this study may limit the statistical power for statistical analyses of gene-covariate interactions. Third, future studies will need to address these polymorphisms with other exposure risk factors of ESCC such as nutritional deficiencies and nitrosamines.

In summary, we found that *CXCR4* rs6430612, *TERT* rs13172201 and *OBFC1* rs4387287 are key genetic components in complicated regulation of telomere length. In accord with this notion, these polymorphisms also significantly contribute to susceptibility to ESCC. Our results elucidate the prevalent involvement of genetic variants in telomere biology and further provide pathogenic insights into the role of telomere in cancer development.

## Supplementary Material

Supplementary table S1.

## Figures and Tables

**Table 1:** Association between telomere length-related genetic variations from previously published studies with leucocyte telomere length in heathy subjects of Jiangsu discovery set

Genes	SNP IDs	Location^1^	Alleles	β-value^1^	SE	*P*^2^	Reference
*CXCR4*	rs6430612	Chromosome 2:137006198	C/T	-0.112	0.061	0.044	Levy D, et al., 2010
*TERT*	rs13172201	Chromosome 5:1271661	C/T	0.125	0.056	0.022	Bao Y, et al., 2017
*TERT*	rs10069690	Chromosome 5:1279790	C/T	-0.069	0.063	0.183	Julin B, et al., 2015
*TERT*	rs2853676	Chromosome 5:1288547	C/T	-0.023	0.053	0.668	Julin B, et al., 2015
*TERT*	rs451360	Chromosome 5:1319680	C/A	0.143	0.047	0.036	Bao Y, et al., 2017
*OBFC1*	rs4387287	Chromosome 10:105677897	A/C	-0.134	0.051	0.011	Levy D, et al., 2010
*VPS34*	rs2162440	Chromosome 18:35214006	G/A	0.013	0.029	0.782	Mangino M, et al., 2009

Note: SNP, single nucleotide polymorphism. 1: Reference genome GRCh37.p13. 2: The association of SNPs with telomere length was assessed using linear regression adjusted for age sex, smoking and drinking status.

**Table 2:** Genotype frequencies of *CXCR4* rs6430612 genetic variant among patients and controls and their association with ESCC risk

	Genotypes	Patients No. (%)	Controls No. (%)	OR^1^ (95% CI)	*P*-value
**Jiangsu set**		*n* = 588	*n* = 600		
	CC	549(93.4)	517(86.2)	Reference	
	CT	37(6.3)	78(13.0)	0.40(0.25-0.62)	4.7×10^-5^
	TT	2(0.3)	5(0.8)	N.C.	N.C.
**Shandong set**		*n* = 997	*n* = 1000		
	CC	911(91.4)	850(85.0)	Reference	
	CT	84(8.4)	139(13.9)	0.57(0.42-0.78)	4.0×10^-4^
	TT	2(0.2)	11(1.1)	N.C.	N.C.
**Total**		*n* = 1585	*n* = 1600		
	CC	1460(92.1)	1367(85.4)	Reference	
	CT	121(7.6)	217(13.6)	0.51(0.40-0.66)	1.7×10^-7^
	TT	4(0.3)	16(1.0)	N.C.	N.C.

Abbreviations: ESCC, esophageal squamous cell carcinoma; OR, odds ratio; CI, confidence interval; N.C., not calculated. 1: Data were calculated by logistic regression with adjustment for age, sex, smoking and drinking status.

**Table 3:** Genotype frequencies of *TERT* rs13172201 genetic variant among patients and controls and their association with ESCC risk

	Genotypes	Patients No. (%)	Controls No. (%)	OR^1^ (95% CI)	*P*-value
**Jiangsu set**		*n* = 588	*n* = 600		
	CC	367(62.4)	432(72.0)	Reference	
	CT	199(33.8)	158(26.3)	1.42(1.08-1.88)	0.012
	TT	22(3.7)	10(1.7)	1.44(0.93-2.21)	0.102
**Shandong set**		*n* = 1000	*n* = 1000		
	CC	623(62.3)	713(71.3)	Reference	
	CT	329(32.9)	267(26.7)	1.33(1.08-1.64)	0.007
	TT	48(4.8)	20(2.0)	1.52(1.14-2.02)	0.004
**Total**		*n* = 1588	*n* = 1600		
	CC	990(62.3)	1145(71.6)	Reference	
	CT	528(33.2)	425(26.6)	1.37(1.16-1.61)	2.2×10^-4^
	TT	70(4.4)	30(1.9)	1.50(1.19-1.91)	0.001

Abbreviations: ESCC, esophageal squamous cell carcinoma; OR, odds ratio; CI, confidence interval. 1: Data were calculated by logistic regression with adjustment for age, sex, smoking and drinking status.

**Table 4:** Genotype frequencies of *OBFC1* rs4387287 genetic variant among patients and controls and their association with ESCC risk

	Genotypes	Patients No. (%)	Controls No. (%)	OR^1^ (95% CI)	*P*-value
**Jiangsu set**		*n* = 588	*n* = 600		
	CC	469(79.8)	422(70.4)	Reference	
	CA	115(19.5)	164(27.3)	0.74(0.55-0.99)	0.049
	AA	4(0.7)	14(2.3)	N.C.	N.C.
**Shandong set**		*n* = 1000	*n* = 1000		
	CC	776(77.6)	707(70.7)	Reference	
	CA	213(21.3)	268(26.8)	0.67(0.54-0.84)	3.9×10^-4^
	AA	11(1.1)	25(2.5)	0.64(0.44-0.95)	0.025
**Total**		*n* = 1588	*n* = 1600		
	CC	1245(78.4)	1129(70.6)	Reference	
	CA	328(20.7)	432(27.0)	0.67(0.58-0.82)	3.9×10^-5^
	AA	15(0.9)	39(2.4)	0.59(0.43-0.82)	0.002
					

Abbreviations: ESCC, esophageal squamous cell carcinoma; OR, odds ratio; CI, confidence interval; N.C., not calculated. 1: Data were calculated by logistic regression with adjustment for age, sex, smoking and drinking status.

**Table 5:** Risk of ESCC associated with *CXCR4*, *TERT* and *OBFC1* SNPs by age, sex, smoking, and drinking status

Variables	*CXCR4* rs6430612						*TERT* rs13172201				
	CC^1^	CT+TT^1^	OR^2^ (95% CI)	*P*	*P*_interaction_		CC^1^	CT+TT^1^	OR^2^ (95% CI)	*P*	*P*_interaction_
**Age (year)**					0.073						0.411
≤57	752/667	51/132	0.33(0.23-0.48)	3.8×10^-7^			497/561	307/238	1.33(1.06-1.68)	0.015	
>57	708/700	74/101	0.69(0.49-0.97)	0.031			493/584	291/217	1.58(1.26-1.99)	8.7×10^-5^	
**Sex**					0.494						0.245
Male	1088/1008	99/181	0.48(0.37-0.64)	2.0×10^-7^			761/851	428/338	1.37(1.14-1.64)	0.001	
Female	372/359	26/52	0.53(0.29-0.99)	0.046			229/294	170/117	1.79(1.24-2.60)	0.002	
**Smoking status**					0.461						0.024
Nonsmoker	366/862	32/139	0.57(0.38-0.86)	0.007			245/740	154/261	1.82(1.41-2.33)	2.8×10^-6^	
Smoker	1094/505	93/94	0.48(0.36-0.66)	4.9×10^-6^			754/405	444/194	1.20(0.97-1.49)	0.090	
**Alcohol drinking**					0.641						0.747
No	674/815	53/142	0.52(0.36-0.75)	4.1×10^-4^			450/697	251/260	1.49(1.18-1.88)	0.001	
Yes	813/552	72/91	0.50(0.36-0.70)	6.3×10^-5^			540/448	347/195	1.40(1.12-1.75)	0.004	

Abbreviations: ESCC, esophageal squamous cell carcinoma; OR, odds ratio; CI, confidence interval. 1: Number of ESCC patients with genotype/number of control subjects with genotype(s). 2: Data were calculated by logistic regression, adjusted for sex, age, smoking, and drinking status, where it was appropriate.

**Table 6:** Risk of ESCC associated with *CXCR4*, *TERT* and *VPS34* SNPs by age, sex, smoking, and drinking status (continued)

Variables	*OBFC1* rs4387287				
	CC^1^	CA+AA^1^	OR^2^ (95% CI)	*P*	*P*_interaction_
**Age (year)**					0.969
≤57	623/563	181/236	0.64(0.50-0.82)	4.8×10^-4^	
>57	622/566	162/235	0.63(0.50-0.81)	3.1×10^-4^	
**Sex**					0.001
Male	946/820	243/329	0.57(0.47-0.70)	2.3×10^-7^	
Female	299/309	100/102	1.08(0.72-1.62)	0.723	
**Smoking status**					0.002
Nonsmoker	285/737	114/264	1.09(0.84-1.42)	0.503	
Smoker	960/392	229/207	0.44(0.35-0.55)	1.5×10^-7^	
**Alcohol drinking**					2.2×10^-4^
No	512/706	189/251	1.06(0.83-1.35)	0.661	
Yes	733/423	154/220	0.40(0.32-0.52)	7.6×10^-8^	

Abbreviations: ESCC, esophageal squamous cell carcinoma; OR, odds ratio; CI, confidence interval. 1: Number of ESCC patients with genotype/number of control subjects with genotype(s). 2: Data were calculated by logistic regression, adjusted for sex, age, smoking, and drinking status, where it was appropriate.
