# Supplementary material for: Identification of Leukocyte telomere length-related genetic variants contributing to predisposition of Esophageal Squamous Cell Carcinoma
Source: J Cancer. 2020 Jun 23;11(17):5025–31. doi: 10.7150/jca.45165 (PMC7378929; doi:10.7150/jca.45165)
Supplement: Supplementary file 1 — Supplementary table S1. [file jcav11p5025s1.pdf]

Supplementary Table 1. Distribution of selected characteristics among ESCC cases and controls

Variable	Jiangsu case-control set (Discovery set)			Shandong case-control set (Validation set)		
	Cases	Controls	*P*-value [a]	Cases	Controls	*P*-value[a]
	No. (%)	No. (%)		No. (%)	No. (%)	
	588	600		1000	1000	
Sex			0.678			0.426
Male	413(70.2)	428(71.3)		776(77.6)	761(76.1)	
Female	175(29.8)	172(28.7)		224(22.4)	239(23.9)	
Age (year)[2]			0.725			0.474
≤59(or 56)	288(49.0)	300(50.0)		516(51.6)	500(50.0)	
>59(or 56)	300(51.0)	300(50.0)		484(48.4)	500(50.0)	
Smoking			<0.001			<0.001
No	151(25.7)	397(66.2)		248(24.8)	604(60.4)	
Yes	437(74.3)	203(33.8)		752(75.2)	396(39.6)	
Drinking			<0.001			<0.001
No	254(43.2)	358 (59.7)		447(44.7)	599(59.9)	
Yes	334(56.8)	242(40.3)		553(55.3)	401(40.1)	

Note: ESCC, esophageal squamous cell carcinoma.

[1]Two-sided χ^2 test.

[2]Median ages of cases for Shandong case-control set and Jiangsu case-control set are 56 and 59 years.
